# Supplementary material for: Content-rich biological network constructed by mining PubMed abstracts
Source: BMC Bioinformatics. 2004 Oct 8;5:147. doi: 10.1186/1471-2105-5-147 (PMC528731; doi:10.1186/1471-2105-5-147)
Supplement: Additional File 5 — The original Chilibot query results of the term "long-term potentiation (LTP)" and 22 other terms, limiting the latest references analyzed to the years 1990, 1995, 2000, and 2004. [file 1471-2105-5-147-S5.bz2 › chilibotAdditionalFile5/ltp2004/html/PKC_PKA.html]

 


 **PKC** and **PKA** 
  
Found 1531 abstracts in PubMed,  **30 abstracts were retrieved and analyzed**.  


---

 Search Google  |
 PDF files only 
|  EDU domain only 

---

**Interactive relationship** (e.g. stimulation, inhibition, etc)

- In experiments using inhibitors of phosphorylation and dephosphorylation, the  **PKA**  mediated effect was shown to be regulated by the phosphorylated states modulated by  **PKC**  endogenously.  Ref: 14727526 Nippon Yakurigaku Zasshi, 2003
- Furthermore, application of DHX in the presence of the  **PKC**  inhibitor BiM1 or internal  **PKC**  inhibitory peptide 19 36 resulted in persistent firing of full calcium spike bursts, suggesting that a D1 D5  **PKA**  mechanism switches subthreshold calcium hump potential to fire full calcium spikes, which are eventually turned off by a D1 D5 calcium dependent  **PKC**  mechanism.  Ref: 14715933 J Neurosci, 2004
- It is well established that beta adrenoceptor stimulation activates  **PKA**  and alpha 1 adrenoceptor stimulation activates  **PKC** .  Ref: 14741699 Biochem Biophys Res Commun, 2004
- Back phosphorylation of TnI from mTnI hearts demonstrated that the mutation of the  **PKC**  sites was associated with an enhanced  **PKA**  dependent phosphorylation independent of a change in basal cAMP levels.  Ref: 14726296 Am J Physiol Heart Circ PhysiolAm J Physiol Heart Circ Physiol, 2004
- These results illustrate that  **PKA**  activation in macrophages is able to stimulate  **PKC**  and p38 MAPK, which lead to IKK dependent NF kappaB activation and contribute to the induction of inducible nitric oxide synthase iNOS and IL 6 genes.  Ref: 14751542 Cell Signal, 2004
- ... the  **PKA**  pathway does not seem to be involved ii downstream from  **PKC** , activation of NF kappa B is essential while ERK1 2 MAP kinases, even though activated by Tat, are not directly involved in the pathway signaling leading to TNF alpha production.  Ref: 14708348 J Soc Biol, 2003
- Basolateral PAH transport presumably OAT1 activity appears to be down regulated by activation of protein kinase C  **PKC**  and up regulated via mitogen activated protein kinase MAPK through phospholipase A 2 PLA 2, prostaglandin E 2 PGE 2, cyclic AMP, and protein kinase A  **PKA**  activation.  Ref: 14729155 Biochim Biophys Acta, 2003

- :-)

  **Parallel relationship** (e.g. studied together, co-existance, homology, etc.)

  - In this study, we examined the roles of spinal protein kinase C  **PKC**  and protein kinase A  **PKA**  in the BV induced different types of pain and hypersensitivity in conscious rats.  Ref: 14739559 NeurosignalsNeurosignals, 2003
  - Reversal, by DMSO, of the effects of SAD on MEPM cell growth, on PCNA expression and on all components of the  **PKA** ... pathway suggests that the perturbation of the  **PKA**  pathway by SAD is relevant to its induction of CP in mice.  Ref: 14761683 Toxicol Appl Pharmacol, 2004
  - **PKA**  dependent activation of  **PKC** , p38 MAPK and IKK in macrophage implication in the induction of inducible nitric oxide synthase and interleukin 6 by dibutyryl cAMP.  Ref: 14751542 Cell Signal, 2004
  - In summary, we have shown that ERK1 2 activation by adenosine in cardiomyocytes results from an additive stimulation of A 1, A 2A, and A 3 ARs, which involves G i o proteins,  **PKC** , and tyrosine kinase for A 1 and A 3 ARs, and Gs and  **PKA**  for A 2A ARs.  Ref: 14751870 Br J Pharmacol, 2004
  - We found that 1 BV induced primary heat hypersensitivity could be blocked by intrathecal pre or posttreatment with a  **PKC**  inhibitor, chelerythrine chloride CH, while a  **PKA**  inhibitor, N 2 P bromocinnamylamino ethyl 5 isoquinolinesulfonamide hydrochloride H89, had no effect.  Ref: 14739559 NeurosignalsNeurosignals, 2004
  - Moreover, the A 3 AR response also involves a cAMP  **PKA**  pathway via  **PKC**  activation.British Journal of Pharmacology 2004 14 29 339.  Ref: 14751870 Br J Pharmacol, 2004
  - They were inhibited by H89  **PKA**  inhibitor but not by GF  **PKC**  inhibitor, and were enhanced by papaverine phosphodiesterase inhibitor and by calyculin protein phosphatase inhibitor.  Ref: 14735495 Mol Reprod Dev, 2004
  - A number of the interphase specific high turnover sites were shown to be in vitro phosphorylation sites for  **PKA**  and protein kinase C  **PKC** .  Ref: 14762106 J Cell Sci, 2004
  - This depolarizing state dependent, D1 D5 activated, bi directional switching of soma dendritic L type calcium channels via  **PKA**  dependent potentiation and  **PKC**  dependent suppression may provide spatiotemporal regulation of synaptic integration and plasticity in PFC.  Ref: 14715933 J Neurosci, 2004
  - In monocytes, calcium,  **PKA** , and  **PKC**  pathways are highly implicated in the expression of cytokine genes.  Ref: 14708348 J Soc Biol, 2003
  - This reduction requires postsynaptic calcium elevation and presynaptic  **PKA**  and  **PKC**  dependent modification of slow inactivating K channels.  Ref: 14741106 Neuron, 2004
  - These actions of dBcAMP were diminished by the presence of the inhibitors of protein kinase A  **PKA** , protein kinase C  **PKC** , p38 MAPK and nuclear factor kappa B NF kappaB.  Ref: 14751542 Cell Signal, 2004
  - Thus, cellulose substrata seem to induce cell cell communication through Cx 43 phosphorylation modulated by  **PKA**  and  **PKC** .  Ref: 14751734 Biomaterials, 2004
  - The protein kinase A  **PKA**  inhibitor, H 89 and the protein kinase C  **PKC**  inhibitor, chelerythrine chloride prevented activation by both peptides demonstrating that  **PKA**  and  **PKC**  are part of the signaling pathway.  Ref: 14744818 Br J PharmacolBr J Pharmacol, 2004
  - These results demonstrated that AMPH induced increases in MAP kinase and  **PKC**  are important for induction of both the enhancement in transporter mediated DA release and neurite outgrowth but  **PKA**  was only required for the enhancement in AMPH stimulated DA release.  Ref: 14713310 J Neurochem, 2003
  - Taken together with our previous report however, spinal  **PKC**  and  **PKA**  are likely to be simultaneously involved in the central processes of both PSN and MIH hypersensitivity.  Ref: 14739559 NeurosignalsNeurosignals, 2003
  - The balanol analogues serve to test the model and to highlight differences in the active site local environment of  **PKA**  and  **PKC** .  Ref: 14705934 Biochemistry, 2004
  - Via  **PKA** ,  **PKC**  and p38 MAPK dependent signals, dBcAMP also induced inhibitory subunit of NF kappaB IkappaB degradation, IkappaB kinase IKK activation, nuclear translocation of NF kappaB subunit p65 and its association with the CREB binding protein CBP.  Ref: 14751542 Cell Signal, 2004
  - We investigated the involvement of hippocampal protein kinase A  **PKA** , protein kinase C  **PKC** , calcium calmodulin dependent protein kinases CaMK II, and mitogen activated extracellular signal regulated kinases 1 and 2 Mek 1 2 in the phosphorylation of their downstream targets extracellular signal regulated kinases 1 and 2 Erk 1 2, cAMP response element binding protein CREB, Elk 1, and p90 ribosomal S6 kinase 1 p90Rsk 1.  Ref: 14739006 Behav Brain Res, 2004
  - Erk 1 2, Elk 1, and CREB were identified as common targets of  **PKA** ,  **PKC** , and CaMK II during memory consolidation the time window and sequence of their phosphorylation was specific for the individual kinase.  Ref: 14739006 Behav Brain Res, 2004
  - Treatment of the cells with acute AMPH elicited an increase in the activity of  **PKC**  and MAP kinase but not  **PKA** .  Ref: 14713310 J Neurochem, 2003
  - C57BL 6N mice were injected into the dorsal hippocampus with inhibitors of  **PKA** ,  **PKC** , CaMK II, Mek 1 2, or vehicle before training consisting of a single exposure to a context, tone, and footshock.  Ref: 14739006 Behav Brain Res, 2004
  - Our results demonstrate that OxPAPC treatment activated in a time dependent fashion protein kinase C  **PKC** , protein kinase A  **PKA** , Raf MEK Erk MAP kinase cascade, JNK MAP kinase and transient protein tyrosine phosphorylation in human pulmonary artery endothelial cells HPAEC.  Ref: 14709399 Microvasc Res, 2004
  - Here, we characterize three balanol analogues that inhibit cyclic adenosine monophosphate dependent protein kinase  **PKA**  more specifically and potently than calcium and phospholipid dependent protein kinase  **PKC** .  Ref: 14705934 Biochemistry, 2004
  - Treatment of the plasma membrane with  **PKC**  but not  **PKA**  induced displacement of Munc18 3 from the membrane.  Ref: 14759605 Arch Biochem Biophys, 2004
  - The activity of calcium ATPase, Na, K ATPase, cAMP dependent protein kinase  **PKA** , calcium phospholipid dependent protein kinase  **PKC** , and tyrosine protein kinase TPK were determined.  Ref: 14761484 Zhonghua Lao Dong Wei Sheng Zhi Ye Bing Za Zhi, 2003
  - On the basis of sequence homology to  **PKA** , a preliminary model of the  **PKC**  active site is described.  Ref: 14705934 Biochemistry, 2004
  - It was concluded that hippocampal  **PKA**  and  **PKC**  play crucial roles in one trial fear conditioning.  Ref: 14739006 Behav Brain Res, 2004
  - Although SAD has been shown to affect both protein kinases A  **PKA**  and C  **PKC**  pathways, the relevance of each of these pathways to its CP induction is unknown.  Ref: 14761683 Toxicol Appl Pharmacol, 2004
  - The upregulation of NOR 1 mRNA levels by LDL involves pertusis sensitive G protein coupled receptors, calcium mobilization, protein kinases A  **PKA**  and C  **PKC**  activation, and mitogen activated protein kinase pathways MAPK p44 p42 and p38.  Ref: 14962944 Arterioscler Thromb Vasc BiolArterioscler Thromb Vasc Biol, 2004
  - In a previous study, we demonstrated a stimulatory effect of the synthetic cannabinoid receptor agonist desacetyllevonantradol DALN on calcium flux into N18TG2 neuroblastoma cells, and suggested a dual mechanism one pathway mediated by  **PKA**  and the other one by protein kinase C  **PKC** .  Ref: 14741403 Brain Res Mol Brain Res, 2004
  - Based on the present findings, we proposed that spinal  **PKC**  might be activated during the central processes of primary heat hypersensitivity, while spinal  **PKA**  is likely to be involved in primary mechanical hypersensitivity induced by subcutaneous BV chemical injury.  Ref: 14739559 NeurosignalsNeurosignals, 2004
  - We investigated the role of protein kinases in the induction of these AMPH mediated events by using inhibitors of protein kinase C  **PKC** , mitogen activated protein kinase MAP kinase or protein kinase A  **PKA** .  Ref: 14713310 J Neurochem, 2003
